# Identification and characterization of DNA endonucleases in *Plasmodium falciparum* 3D7 clone

**DOI:** 10.1186/s12936-018-2388-0

**Published:** 2018-06-18

**Authors:** Ning Jiang, Zhiwei Tu, Yiwei Zhang, Jianping Li, Ying Feng, Na Yang, Xiaoyu Sang, Qijun Chen

**Affiliations:** 1 Key Laboratory of Zoonosis, College of Animal Science and Veterinary Medicine, Shenyang Agricultural University, 120 Dongling Road, Shenyang, 110866 People’s Republic of China; 2 Key Laboratory of Zoonosis, Ministry of Education, Institute of Zoonosis, Jilin University, Changchun, 130062 People’s Republic of China; 3 Blood Center of Liaoning Province, 13 Beihai Street, Shenyang, 110866 People’s Republic of China

**Keywords:** Malaria, *Plasmodium falciparum*, DNA endonuclease, Catalysis

## Abstract

**Background:**

*Plasmodium falciparum* is the most virulent parasite of the five *Plasmodium* species that cause human malaria, and biological analysis of the parasite is critical for the development of novel strategies for disease control. DNA endonucleases are important for maintaining the biological activity, gene stability of the parasite and interaction with host immune systems. In this study, ten sequences of DNA endonucleases were found in the genome of *P. falciparum* 3D7 clone, seven of them were predicted to contain an endonuclease/exonuclease/phosphatase (IPR005135) domain which plays an important role in DNA catalytic activity. The seven DNA endonucleases of *P. falciparum* were systematically investigated.

**Methods:**

*Plasmodium falciparum* 3D7 clone was cultured in human O^+^ RBCs, RNA was extracted at 8, 16, 24, 32, 40, and 48 h post invasion and real-time quantitative PCR was carried out to analyse the transcription of the seven DNA endonuclease genes in asexual stages. Immunofluorescence assay was carried out to confirm the location of the encoded proteins expressed in the erythrocytic stages. Finally, the catalytic activity of the DNA nucleases were tested.

**Results:**

Of the seven proteins analysed, two proteins were not soluble. Fragments derived from the rest five endonuclease sequences were successfully expressed as soluble proteins, and which were used to generate antisera for protein localization. The proteins were all located in the nucleus at ring and trophozoite stages. While at schizont stage, proteins encoded by PF3D7_1238600, PF3D7_0107200 and PF3D7_0319200 were in the punctuated forms in the parasite most likely around nuclei of the merozoites. But the proteins encoded by PF3D7_0305600 and PF3D7_1363500 were distributed around the infected erythrocyte membrane. The enzymatic activity of the recombinant GST-PF3D7_1238600 was very efficient without divalent iron, while the activity of the rest four enzymes was iron dependent. Further, divalent irons did not show any specific enhancement on the activity of GST-PF3D7_1238600, but the activity of GST-PF3D7_0107200, GST-PF3D7_1363500 and GST-PF3D7_0319200 were Cu^2+^ dependent. The activity of GST-PF3D7_0305600 was dependent on Mg^2+^ and Mn^2+^. Except GST-PF3D7_1363500, four of the GST tagged recombinant proteins hydrolysed the supercoiled circular plasmid DNA with or without divalent metal ions. The GST-PF3D7_1363500 protein only changed the supercoiled circular plasmid DNA into nicked plasmids, even with Cu^2+^.

**Conclusions:**

Fragments derived from five of the endonuclease sequences of *P. falciparum* 3D7 clone were successfully expressed. The proteins displayed diverse cell distribution, biochemical and enzymatic activities, which indicated that they carried different biological function in the development of the parasite in the erythrocytes. The DNA repair and DNA degradation capacity of the DNA endonucleases in the biology of the parasite remained further studied.

**Electronic supplementary material:**

The online version of this article (10.1186/s12936-018-2388-0) contains supplementary material, which is available to authorized users.

## Background

*Plasmodium falciparum* is the most virulent parasite of all five *Plasmodium* species that cause human malaria, an estimated 3.3 billion people are at risk of malaria, and 1.2 billion are at high risk [1]. The main pathophysiological symptoms of malaria are caused by repeated merozoite invasion into RBCs and exponential parasite proliferation in the blood stage.

DNA endonucleases are a type of enzymes that hydrolyse internal phosphodiester bonds, which exist in DNA strands. DNase I is a DNA-specific enzyme that was discovered in the cells of spleen, liver and digestive tracts of mammalian hosts [2]. Some pathogens successfully survive from the killing of the host cells by the expression of DNases which can degrade the neutrophil extracellular traps (NETs) [3–5]. While NETs are mainly composed of DNA and proteases which released from neutrophils and contributed to the innate immune response by capturing pathogens [6, 7]. Further, it was reported that hosts infected with *Plasmodium malariae*, was accompanied by increased DNase and RNase activities in the sera [8]. During the necrocytosis, DNase I and the plasma fibrinolysis system concentrate at the nucleus of the dead cell and degrade chromosomal DNA, which prevents the appearance of anti-nucleus antibodies [9]. DNase II is a type of acid endonuclease that is independent of divalent metal ions. In mouse fetal development, a deficiency of DNase II leads to the accumulation of large DNA-containing bodies that were resulted from engulfed, but undigested cell corpses in tissues, such as thymus, kidney, spleen, and liver, which could result in dyserythropoietic anaemia and death of the fetus [10]. Deficiency of DNase II in adult mice results in chronic polyarthritis [11]. Apoptotic DNA leads to cell cycle arrest of fibroblasts and epithelial cells. Degraded apoptotic DNA by DNase II activated p53 and p21 pathways, which protected normal cells from apoptotic DNA [12].

The function of DNases is mostly determined by endonuclease/exonuclease/phosphatase (EEP) domain [13–20]. EEP hydrolyses the phosphodiester bond in nucleic acids, proteins and phospholipids. The EEP domain exists in a large number of enzymes, including AP endonuclease, DNase I, inositol-polyphosphate 5-phosphatase and sphingomyelinase, and these enzymes participate in DNA metabolic processes and intracellular signalling [14, 15].

*Plasmodium falciparum* contains a 23 Mb nuclear genome encoding 5400 genes on 14 linear chromosomes [21], a 35 Kb apicoplast genome [22] and a 6 Kb mitochondrion genome [23]. Over 50% of the genes’ encoded proteins have not been well studied [21, 24, 25]. Here, proteins with EEP domains that may encompass DNA hydrolytic ability of *P. falciparum* 3D7 clone were identified and characterized. This study combined a bioinformatics assessment, protein localization and DNA catalytic activity tests. The data generated will facilitate a better understanding of the biology of *P. falciparum*.

## Methods

### Parasites and culture

Parasites of *P. falciparum* 3D7 clone [26] were cultured in human O^+^ RBCs as previously described [27] and synchronized [28] with 5% sorbitol at early ring stage post-invasion. Parasites were harvested at 8, 16, 24, 32, 40 and 48 h post-invasion.

### Sequence and bioinformatic analysis

Ten sequences of DNA endonucleases were found in the genome of *P. falciparum* 3D7 clone (http://plasmodb.org/plasmo/), seven of them contain an EEP domain respectively. The amino acid and nucleotide sequences of the seven genes of *P. falciparum* 3D7 clone were downloaded at PlasmoDB (http://plasmodb.org/plasmo/). Domain prediction and classification were conducted with InterPro (http://www.ebi.ac.uk/interpro/) and SCOP (http://scop.mrc-lmb.cam.ac.uk/scop/index.html). Homologous sequences of other species were downloaded at National Center for Biotechnology Information (NCBI) (http://www.ncbi.nlm.nih.gov/). DNAMAN (version 6.0.3.99) was used for molecular weight calculation and analysis of recombinant protein solubility. Multiple sequence alignments and phylogenetic analysis were conducted with COBALT (http://www.st-va.ncbi.nlm.nih.gov/tools/cobalt/re_cobalt.cgi) and MEGA 6.

### Preparation of cDNA and real-time quantitative PCR

Parasite RNA at six time points post invasion was extracted by TRIzol Reagent (Invitrogen, Carlsbad, CA, USA) according to the manufacturer’s instructions. DNA was removed by DNase I (TaKaRa, Dalian, China), and AMV reverse transcriptase (TaKaRa, Dalian, China) and oligo(dT) primer (TaKaRa, Dalian, China) were used to obtain first-strand cDNA. Real-time quantitative PCR was carried out as previously described [29]. The seryl-tRNA synthetase gene (PF3D7_1205100) is stably transcribed in blood stage, and was used as the internal control [30]. The primers for real-time quantitative PCR are listed in Table 1. Real-time quantitative PCR was conducted on an ABI PRISM^®^ 7500 Real-Time PCR System (Applied Biosystems, CA, USA) with SYBR^®^ Premix Ex Taq™ (TaKaRa). Transcription changes were calculated as 2^−ΔΔCt^ [31]. The mean and standard error were determined using three biological and technical replicates.

**Table 1:** Primers in real time PCR

Genes ID	Forward primer	Reverse primer
PF3D7_1238600	TTCCGGATGTATTTTGTTCC	CCCATTTGCTTAATTCATCG
PF3D7_0107200	CAGTTTATCCTTTTGAATATAATTAT	TGTTCAGGAGTATGTAAGATAAAAT
PF3D7_1363500	AGAAAACAACTTAATGCTATGTC	TGTCGAAAGTGGGTAAATTG
PF3D7_0305600	TAGACACTCAGGAATCGCAAGAAT	CCATAAGTTTGGTTTCTTTGTGAC
PF3D7_0319200	ACGGCTAGCCAAAGTAAC	GAATTCTGTTCCTCGTTTTT
PF3D7_0519500	AGTCCGGATATAGTATGTTTGC	CCCTCCTCTCCTTTTTCCT
PF3D7_1430600	AGCTGGAATGTAAATGGTTG	ACTGATTCGTTCGTTTTGGT

### Expression and purification of His-tagged and GST-tagged recombinant proteins

Specific primers were designed for amplification of the genes and expression of His-tagged and GST-tagged recombinant proteins (Tables 2 and 3) in the plasmids pET-28a and pGEX-4T-1, respectively. *Escherichia coli* BL21 (DE3) strain was used for the generation of the recombinant proteins which were purified with His-Trap purification kit (GE, USA) and glutathione-Sepharose, respectively [32].

**Table 2:** Primers in construction of His-tagged recombinant proteins and expression plasmid

Genes ID	Primers		Recombinant plasmid
PF3D7_1238600	Forward primer	GGATCC TATGATATTGATATATTAGTTCT	pET-28a
	Reverse primer	CTCGAG ATTTGCAACTAGGATATAATCTA	
PF3D7_0107200	Forward primer	GGATCC TTTAATAGCGCTTGGTATACA	pET-28a
	Reverse primer	CTCGAG ATGATCTGATGGGAAATGCTC	
PF3D7_1363500	Forward primer	GGATCC ATACCTTCTGTGCGTAATATAAA	pET-28a
	Reverse primer	CTCGAG TAAGAGCTCAAATACTTTGG	
PF3D7_0305600	Forward primer	GGATCC AATAATAATTCTCCCTTTAGTCA	pET-28a
	Reverse primer	CTCGAG TAAACTAAAATAAGCATTATAATC	
PF3D7_0319200	Forward primer	GGATCC AAAAGTTATACATTTCCATATATA	pET-28a
	Reverse primer	TCTGAG TAAAAGCTTTAATTCCTTGTGAT

**Table 3:** Primers in construction of GST-tagged recombinant proteins and expression plasmid

Genes ID	Primers		Recombinant plasmid
PF3D7_1238600	Forward primer	GGATCC TATGATATTGATATATTAGTTCT	pGEX-4T-1
	Reverse primer	CTCGAG ATTTGCAACTAGGATATAATCTA	
PF3D7_0107200	Forward primer	GGATCC TTTAATAGCGCTTGGTATACA	pGEX-4T-1
	Reverse primer	CTCGAG ATGATCTGATGGGAAATGCTC	
PF3D7_1363500	Forward primer	GGATCC AAGGAATTTTCCGTTTTCTCTTT	pGEX-4T-1
	Reverse primer	CTCGAG GTACGAATAAAATATATAATCTACAC	
PF3D7_0305600	Forward primer	GGATCC ATTATTGTTACATGGAATATGAAT	pGEX-4T-1
	Reverse primer	CTCGAG TTCATTTTTGAGGTATAATATAAC	
PF3D7_0319200	Forward primer	GGATCC CGTATTCTATCGTATAATATTTTAGCA	pGEX-4T-1
	Reverse primer	CTCGAG TAATTCTACCTCAGCAGCTATG	

### Generation of specific antibodies and detection of native proteins in Western blots

To obtain a specific antiserum, 300 μg of His-tagged recombinant protein emulsified with Freund’s Adjuvants was injected into female New Zealand white rabbits every 2 weeks. After four injections, the antiserum and purified total IgG were collected with Protein A Sepharose™ 4 Fast Flow (GE Healthcare) according to the manufacturer’s protocol. Western blot was carried out for detection of native proteins. Erythrocytes infected with parasites were isolated by centrifugation with gradient Percoll (GE health) as described [33] and then lysed in the loading buffer containing 250 mM Tris, 1.92 M glycine and 1% SDS. The proteins were resolved in SDS-PAGE gel and transferred on a nylon membrane. The rabbit anti-His-tagged recombinant protein IgG (1 mg/ml) was used as a primary antibody (1:500). Alkaline phosphatase conjugated goat anti-rabbit IgG (Sigma, 1:10,000) was used as a secondary antibody. The membrane was developed with BCIP/NPT substrate (sigma) to reveal native proteins.

### Immunofluorescence assay

Indirect immunofluorescence assays (IFA) were carried out to localize the proteins inside the parasites. Thin smears with parasites at ring, trophozoite and schizont stages were made and then fixed with 4% paraformaldehyde containing 1 × PBS, 0.8 M NaOH and 0.0075% glutaraldehyde for 15 min. Next, parasite membrane was permeabilized with 0.01% TritonX-100 for 15 min. The slides were blocked with TBST containing 5% non-fat milk (Sigma, St. Louis, USA) for 1 h, and incubated with the protein-specific antibody mentioned above (with a dilution of 1:100 for anti-PF3D7_0305600 IgG and anti-PF3D7_1363500 IgG; 1:50 for anti-PF3D7_1238600 IgG; 1:25 for anti-PF3D7_0107200 IgG and anti-PF3D7_0319200 IgG). The secondary antibody [1:1000, Alexa Fluor 488-conjugated goat anti-rabbit IgG (Invitrogen)] and Hoechst 33342 (Invitrogen, USA) were added. A fluorescence microscope (Olympus, BX 53) was used for capturing high resolution images.

### DNA catalytic assay

A DNA catalytic assay was carried out in a 10 µl volume solution in 1 × PBS, with 10 ng genomic DNA extracted from *P. falciparum* 3D7 clone as previously described [33] and 1.6 μg recombinant protein. The GST protein was included as a negative control. The reaction was conducted at 37 °C for 5, 10, 15, 30, 45, and 60 min. Then, the hydrolysed DNA was detected via agarose gel electrophoresis.

To test the dependency of ion on the enzymatic activity, divalent metal ions (Cu^2+^, Mn^2+^, Ca^2+^, Ni^2+^, Mg^2+^, Co^2+^, and Zn^2+^) were added to the reaction respectively with DNA in linear or circular form as described above. Agarose gel electrophoresis was used for detection of the digested DNA.

## Results

### Sequence and EEP domain identification

**Fig. 1:**
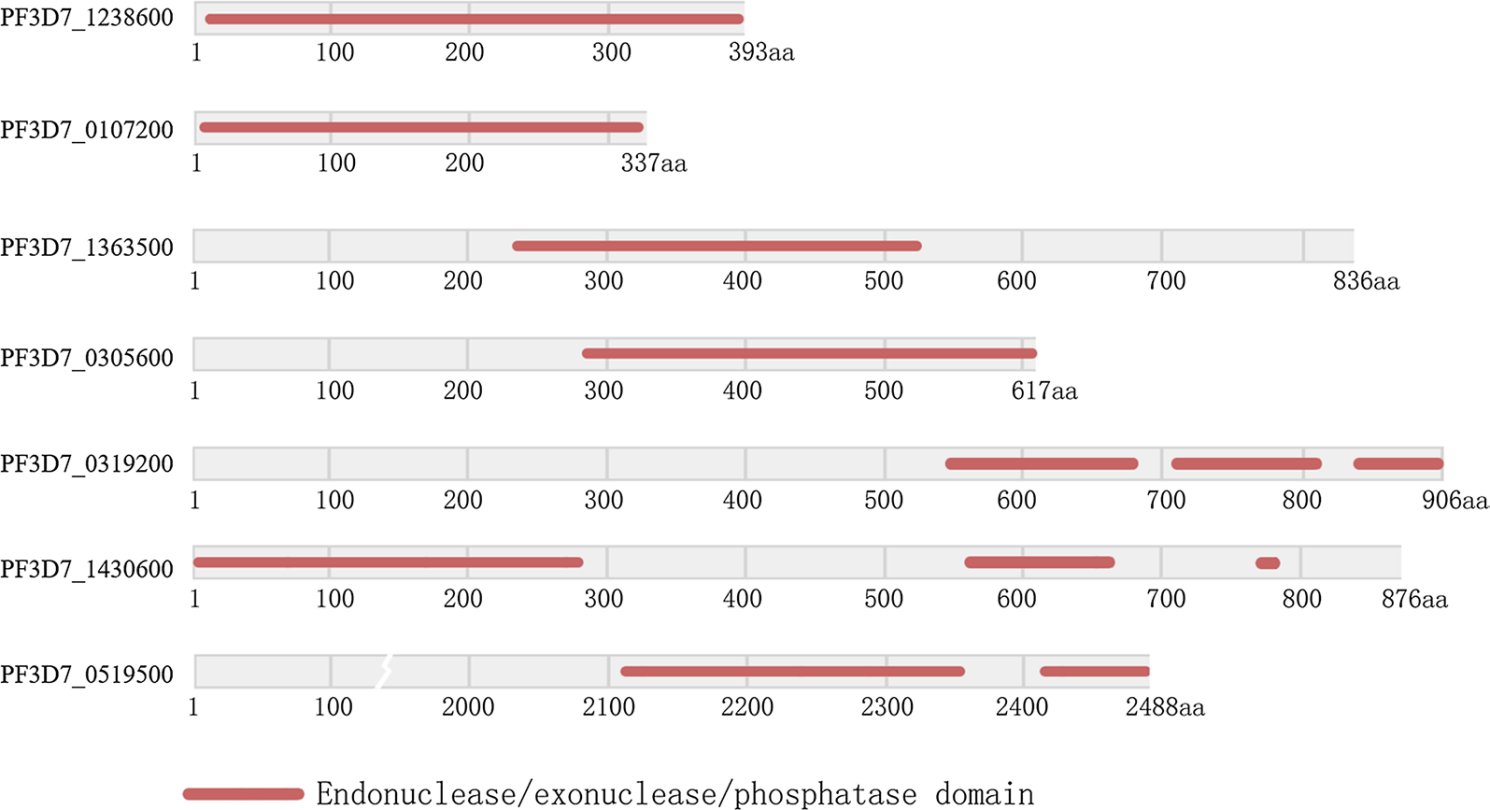
Schematic map of EEP domain within seven DNA endonucleases of *P. falciparum* 3D7 clone. Grey indicates full length of proteins, and red indicates the EEP domain analysed with InterPro

Seven genes encoding proteins with an endonuclease/exonuclease/phosphatase (IPR005135) (EEP) were identified in the genome of *P. falciparum* 3D7 clone (Fig. 1). All identified proteins belong to the DNase I-like superfamily according to structure identification in proteins (SCOP). The homologous sequences of PF3D7_1363500 were found in *Theileria orientalis* strain Shintoku, *Theileria parva* and *Babesia microti* strain RI. The homologous sequence of protein PF3D7_0519500 was found in *Cryptosporidium muris* RN66. Homologous sequences of protein PF3D7_1430600 were in *Trypanosoma vivax* Y486 and *Vitrella brassicaformis* CCMP3155 (see Additional file 1).

### Transcription analysis

**Fig. 2:**
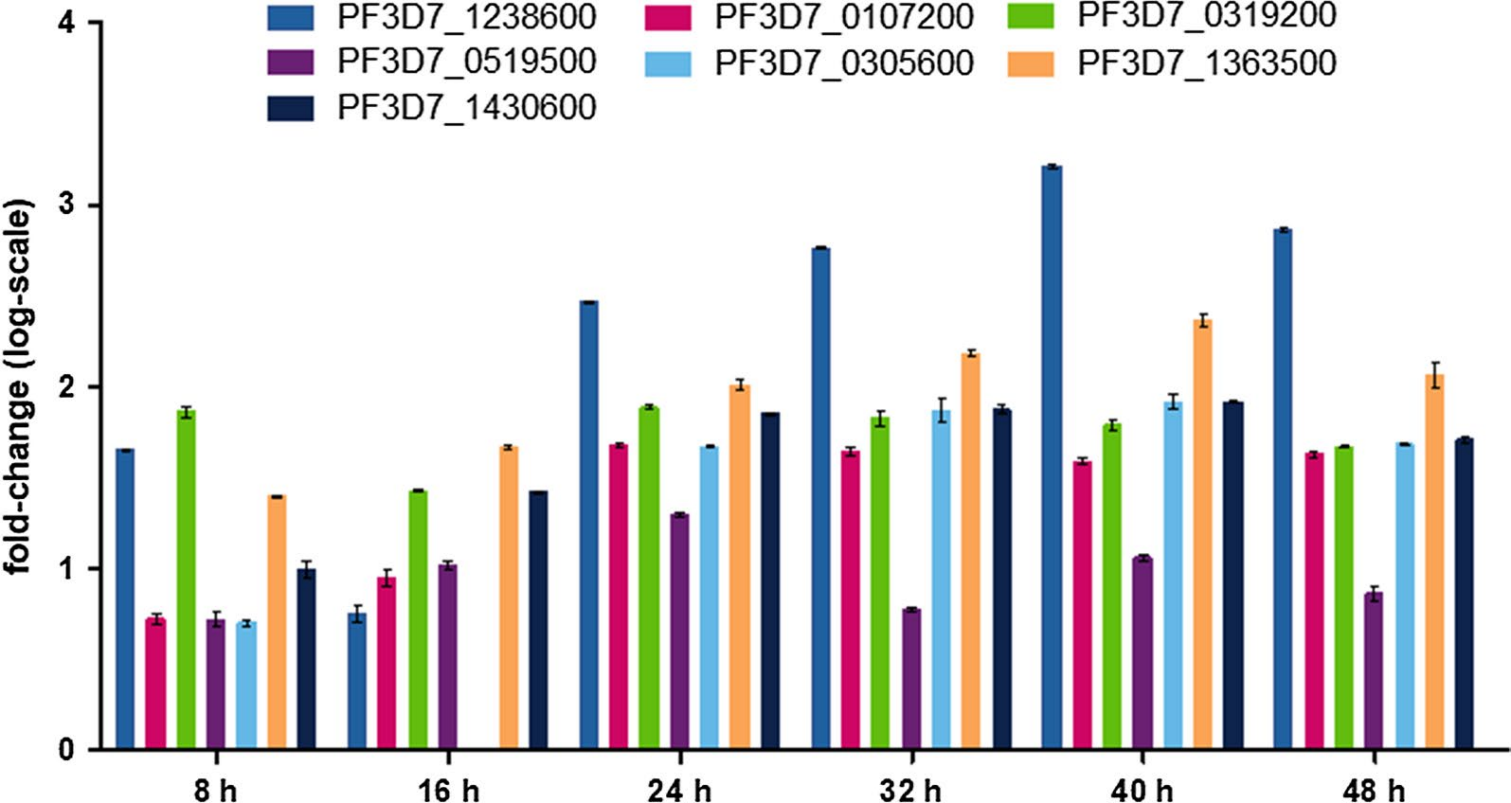
Transcription of the seven DNA endonucleases genes of the *P. falciparum* 3D7 clone. The transcriptions of the seven DNA endonuclease genes at 8, 16, 24, 32, 40 and 48 h post invasion are shown. Transcript levels relative to that of gene PF3D7_0305600 at 16 h post invasion were calculated as 2^−∆∆Ct^. A log-scale was calculated and used on the y-axis

In qPCR, all seven genes were found transcribed at the six time points post erythrocyte invasion. Gene PF3D7_1238600 showed the highest transcriptional level and gene PF3D7_0519500 showed the lowest transcriptional level of the seven genes at the time points of 24, 32, 40 and 48 h. The transcription is generally higher when the parasites reach more mature stage, after 16 h post erythrocyte invasion (Fig. 2).

### Expression and purification of His-tagged and GST-tagged recombinant proteins

Of the seven protein analysed, two proteins encoded respectively by PF3D7_1430600 and PF3D7_0519500 were not soluble. His-tagged and GST-tagged recombinant proteins (see Additional files 2 and 3) were generated and verified by SDS-PAGE and Western blot.

### Detection of native proteins by Western blot and IFA

**Fig. 3:**
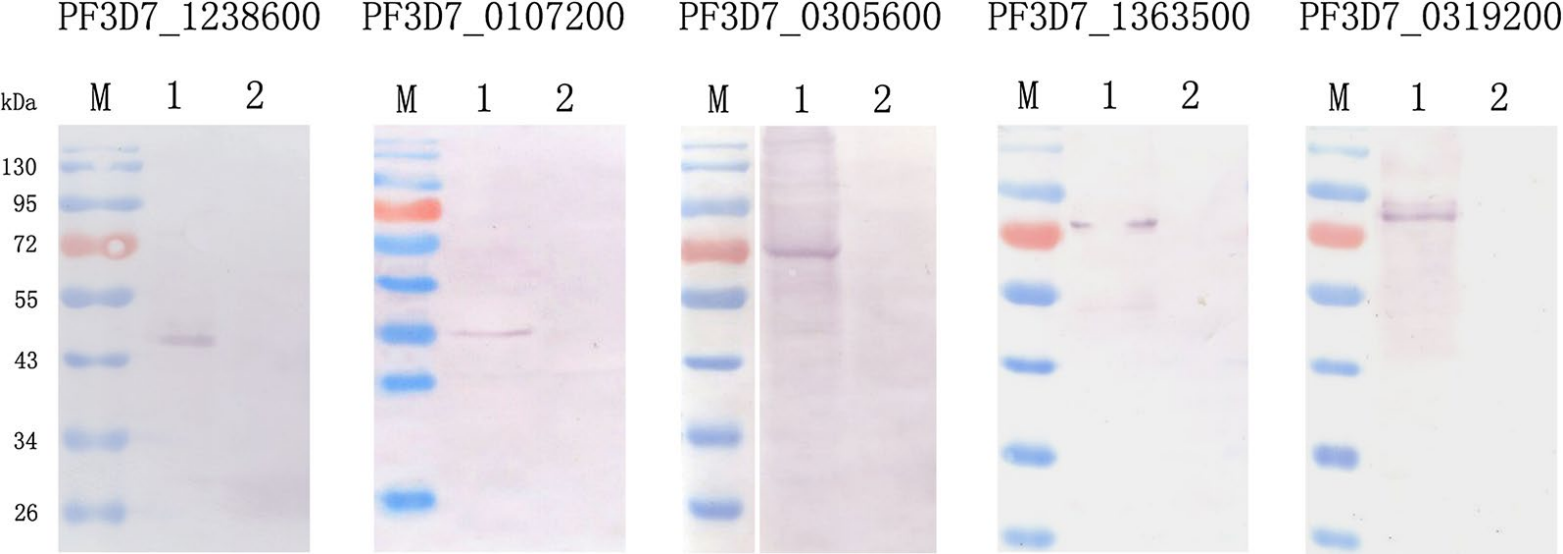
Western blot analysis of the native DNA endonucleases in the *P. falciparum* 3D7 clone. Lane 1 represents infected erythrocytes, and lane 2 represents uninfected erythrocytes. A single band was detected in infected erythrocytes. Protein specific IgG was used as a primary antibody. Alkaline phosphatase conjugated goat anti-rabbit IgG was used as a secondary antibody

Western blot was carried out for the detection of the native proteins in the blood stage of *P. falciparum* 3D7 clone. Protein specific IgGs generated from rabbits were used as primary antibodies. The molecular weight of the protein displayed in the Western blot was consistent with bioinformatic prediction (Fig. 3).

**Fig. 4:**
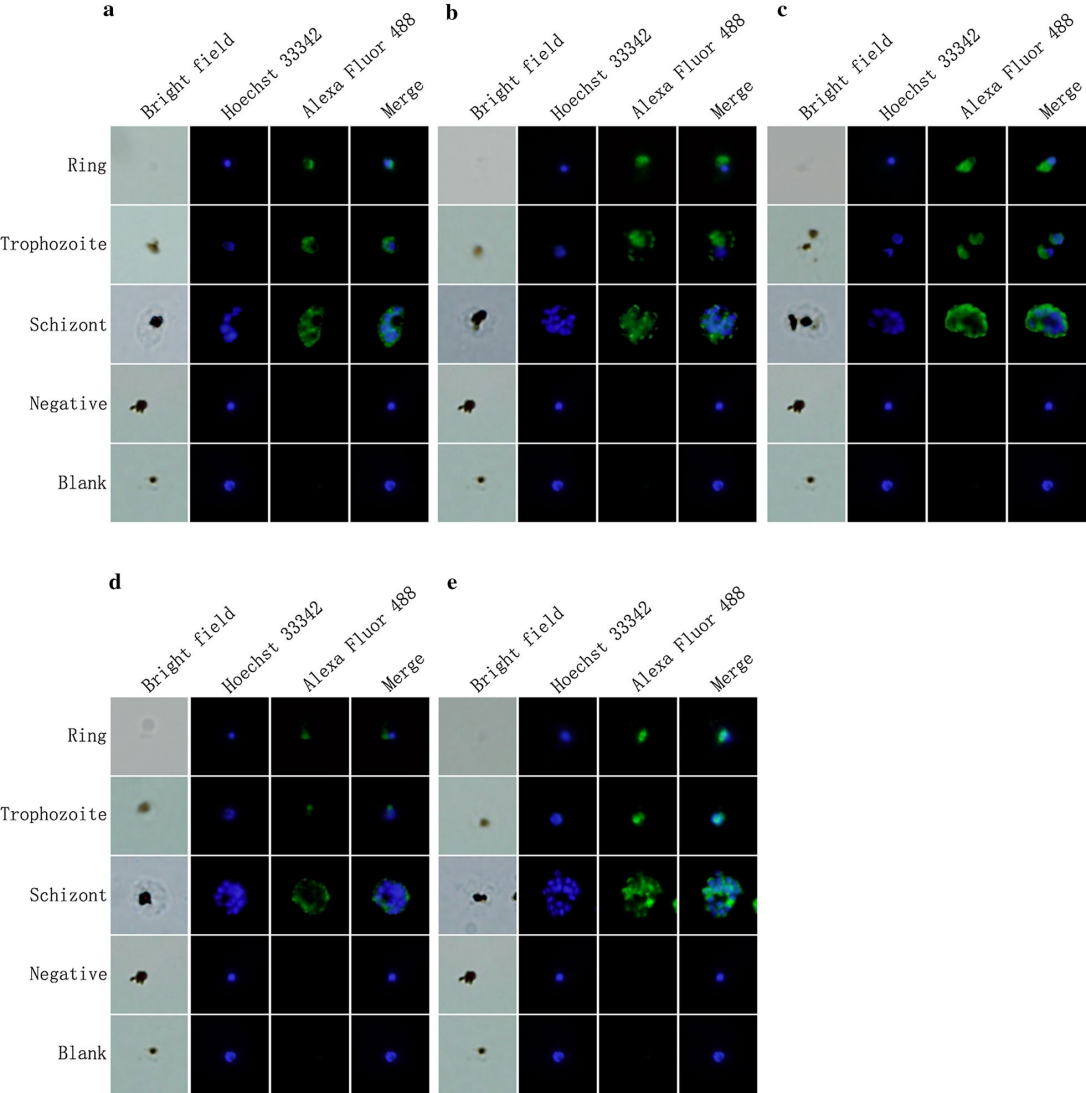
Localization of the five DNA endonucleases in the *P. falciparum* 3D7 clone by immunofluorescence assays. Immunofluorescence assay (IFA) was performed with protein-specific IgG as the primary antibody. Alexa Fluor 488-conjugated goat anti-rabbit IgG was used as a secondary antibody. Hoechst 33342 stained the nuclei blue. **a** Rabbit anti-PF3D7_1238600 IgG was used as a primary antibody. **b** Rabbit anti-PF3D7_0107200 IgG was used as a primary antibody. **c** Rabbit anti-PF3D7_0305600 IgG was used as a primary antibody. **d** Rabbit anti-PF3D7_1363500 IgG was used as a primary antibody. **e** Rabbit anti-PF3D7_0319200 IgG was used as a primary antibody

The proteins were further localized by immunofluorescence assay (IFA) in the ring, trophozoite and schizont developmental stages with protein-specific IgG. The proteins were all located in the nucleus at ring and trophozoite stages. While at schizont stage, proteins encoded by PF3D7_1238600, PF3D7_0107200 and PF3D7_0319200 (Fig. 4a, b, e) were in the punctuated forms in the parasite most likely around nuclei of the merozoites. But the proteins encoded by PF3D7_0305600 and PF3D7_1363500 (Fig. 4c, d) were distributed around the infected erythrocyte membrane.

### DNA nuclease activity test

**Fig. 5:**
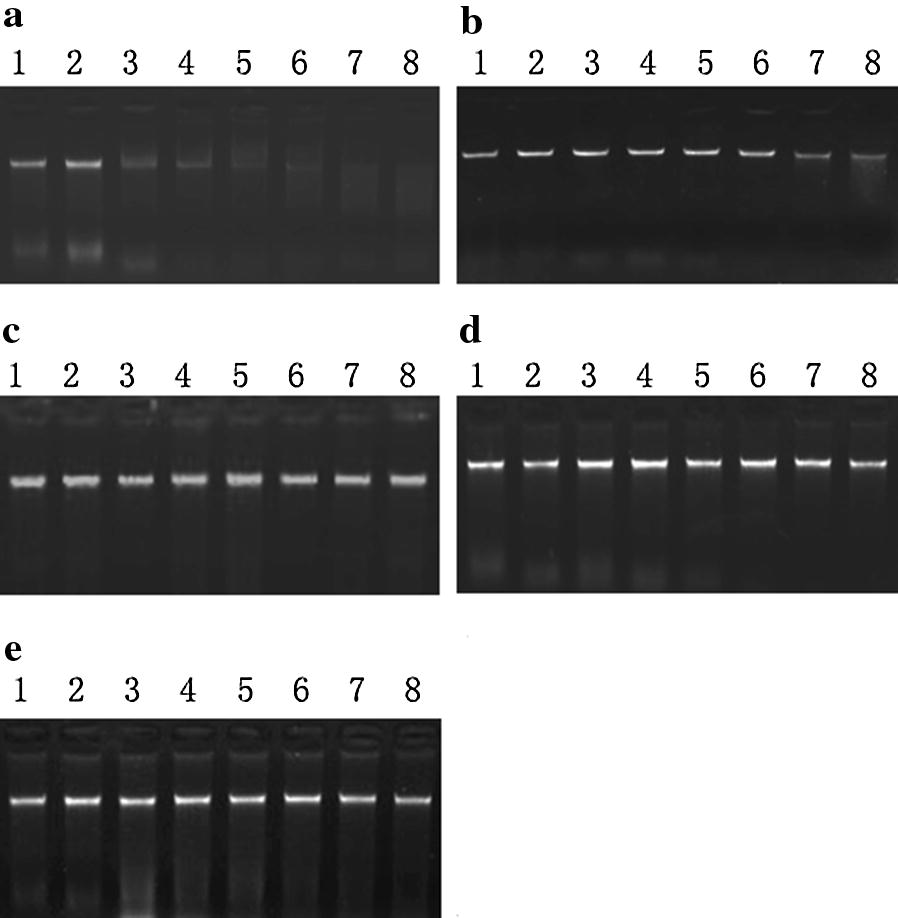
DNA hydrolytic test of the five GST-tagged recombinant proteins without divalent ions. Lane 1 is blank with only genomic DNA incubated at 37 °C for 60 min. Lane 2 is the negative control with genomic DNA and GST protein incubated at 37 °C for 60 min. Lanes 3–8 are genomic DNA and GST-tagged recombinant proteins incubating at 37 °C for 5, 10, 15, 30, 45 and 60 min. **a** GST-PF3D7_1238600 (0.1 mg/ml); **b** GST-PF3D7_0107200 (0.1 mg/ml). **c**. GST-PF3D7_1363500 (0.07 mg/ml); **d** GST-PF3D7_0319200 (0.1 mg/ml); **e** GST-PF3D7_0305600—(0.2 mg/ml)

**Fig. 6:**
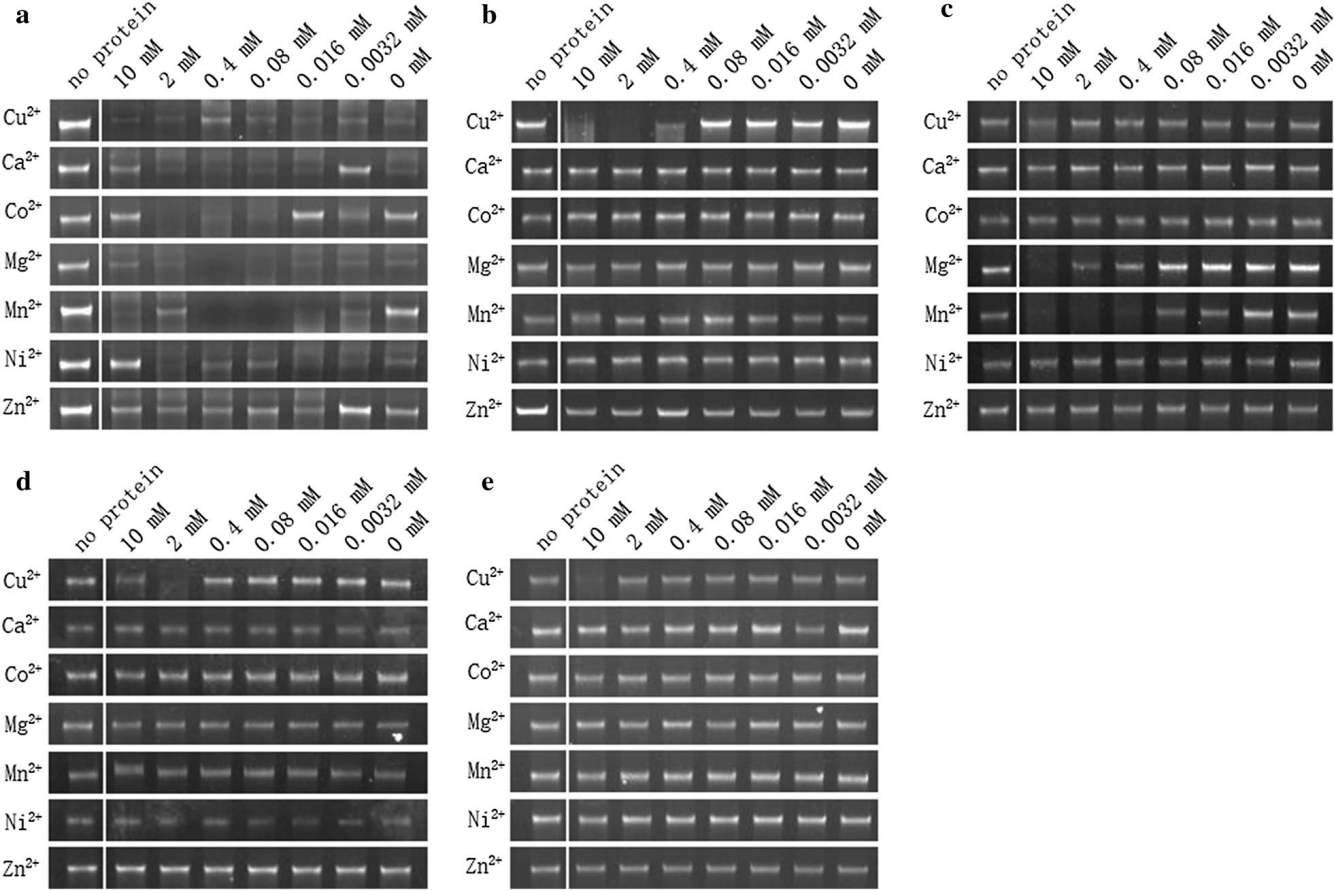
DNA hydrolytic test of the five GST-tagged recombinant proteins with different divalent metals. Divalent metal ions at different concentrations were added into the reaction and incubated for 30 min. The lane with no protein is the blank control with only genomic DNA incubated for 30 min. **a** No specific effect of divalent metal ions on the activity of GST-PF3D7_1238600 was observed. **b** The activity of GST-PF3D7_0107200 was dependent on Cu^2+^ with an optimal concentration of 2 mM. **c** The activity of GST-PF3D7_0305600 was dependent on Mn^2+^ and Mg^2+^. **d** The activity of GST-PF3D7_1363500 was dependent on Cu^2+^ with an optimal concentration of 2 mM. **e** The activity of GST-PF3D7_0319200 was dependent on Cu^2+^ with an optimal concentration of 10 mM

**Fig. 7:**
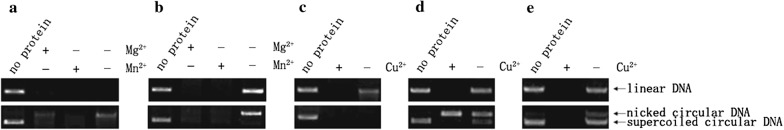
The catalytic effect of the five GST-tagged recombinant proteins on linear DNA and supercoiled circular plasmid DNA. Linear genomic DNA and supercoiled plasmid were used as substrates in a DNA digestion assay. **a** GST-PF3D7_1238600 digestion of linear genomic DNA and supercoiled circular plasmid with or without Mn^2+^ and Mg^2+^. **b** GST-PF3D7_0305600 digestion of linear genomic DNA and supercoiled circular plasmid with Mn^2+^ or Mg^2+^. **c** GST-PF3D7_0107200 digestion of linear genomic DNA and supercoiled circular plasmid with or without Cu^2+^. **d** GST-PF3D7_1363500 digestion linear genomic DNA with Cu^2+^. **e** GST-PF3D7_0319200 digestion of linear genomic DNA and supercoiled circular plasmid with Cu^2+^

The enzymatic activity of the recombinant GST-PF3D7_1238600 was very efficient without divalent iron (Fig. 5a), while the activity of the rest four enzymes were iron dependent (Fig. 5b–e). Further, divalent irons did not show any specific enhancement on the activity of GST-PF3D7_1238600 (Fig. 6a), but the activity of GST-PF3D7_0107200, GST-PF3D7_1363500 and GST-PF3D7_0319200 were Cu^2+^ dependent (Fig. 6b, d, e). The activity of GST-PF3D7_0305600 was dependent on Mg^2+^ and Mn^2+^ (Fig. 6c). Except GST-PF3D7_1363500, four of the GST tagged recombinant proteins hydrolysed the supercoiled circular plasmid DNA with or without divalent metal ions (Fig. 7a–c, e). The GST-PF3D7_1363500 protein only changed the supercoiled circular plasmid DNA into nicked plasmids, even with Cu^2+^ (Fig. 7d).

## Discussion

The function of a protein is closely related to its captured domains. Proteins with the same function share similar domains. In this study, a common domain, EEP domain with activity of hydrolysis of phosphodiester bonds in nucleic acids, proteins and phospholipids was identified in 7 protein sequences of DNases in *P. falciparum*. The EEP domain exists in a large number of enzymes, including AP endonuclease, DNase I, inositol-polyphosphate 5-phosphatase and sphingomyelinase, and these enzymes participate in DNA metabolism processes and intracellular signalling [14, 15].

The DNase I-like superfamily is a member of SCOP 1.75, which groups protein structural domains hierarchically into class, fold, superfamily and family. This superfamily contains three families: DNase I-like, inositol polyphosphate 5-phosphatase and sphingomyelin phosphodiesterase-like. Except the protein PF3D7_1238600, which belongs to the sphingomyelin phosphodiesterase-like family, six of the identified proteins belong to the DNase I-like family. Proteins PF3D7_0305600 and PF3D7_1430600 were AP endonuclease 1 family members in InterPro analysis, and they specifically create a nick at the AP site in the DNA base excision repair pathway. In eukaryotes, there is only one AP endonuclease. However, in *E. coli*, endonuclease IV and exonuclease III are the AP endonucleases [34].

In transcriptional analysis, the lowest transcription level relative to the internal control gene was used for normalization; the fold changes of the gene PF3D7_0305600 relative to the control at 16 h post invasion was set as one. The transcription levels of the genes PF3D7_1238600 and PF3D7_1363500 were respectively a thousand times and a hundred times higher than that of PF3D7_0305600, and the results were consistent with that obtained by microarray assays recorded in PlasmoDB. Peak transcript levels may represent the main stages of activity of the encoded proteins. All seven genes reached their peak transcription at the late trophozoite and early schizont stages, which was further confirmed by Western blot assays (Fig. 3).

The distribution of the proteins inside the infected erythrocytes were mainly in two patterns. The proteins were all located in the nucleus at ring and trophozoite stages. While at schizont stage, proteins encoded by PF3D7_1238600, PF3D7_0107200 and PF3D7_0319200 were in the punctuated forms in the parasite cytoplasm around nuclei of the merozoites (Fig. 4a, b, e). But the proteins encoded by PF3D7_0305600 and PF3D7_1363500 were distributed around the infected erythrocyte membrane (Fig. 4c, d). The phylogenetic analysis indicated that the genes were grouped in separated clusters implying that they perform different function in the development of the parasite.

The DNA catalytic activity of five proteins containing the EEP domain was investigated, and all of the proteins displayed DNA hydrolytic activity with different dependency in divalent irons (Figs. 5, 6 and 7). Thus the proteins with EEP domains encoded by the genes identified in the *P. falciparum* genome indeed could catalyse DNA in a similar manner as observed in other organisms. However, the function of these enzymes in the biology of the parasite remained further investigation.

## Conclusions

Seven genes encoding potential DNA hydrolytic activity were identified in the *P. falciparum* genome and their transcription was analysed by qPCR. The expression of five proteins containing an EEP domain were confirmed by Western blot and IFA, and their DNA catalysis activity were analysed. The proteins displayed diverse cell distribution, biochemical and enzymatic activities, which indicated that they carried different biological function in the development of the parasite in the erythrocytes.

## Additional files

**Additional file 1.** Phylogenetic analysis of seven *P. falciparum* 3D7 DNA endonucleases with homologous proteins of other species. Amino acid sequences were aligned using MEGA 6, and a phylogenetic tree was generated by the neighbour-joining method. The scale bar represents amino acid substitutions in the sequences and evolutionary distances. *P. falciparum* DNA endonucleases are highlighted in red.

**Additional file 2.** Purification of His-tagged recombinant proteins. A. SDS-PAGE analysis of purified His-tagged recombinant proteins. His-tagged proteins of PF3D7_1238600 (Lane 1), PF3D7_0107200 (Lane 2), PF3D7_0305600 (Lane 3), PF3D7_1363500 (Lane 4) and PF3D7_0319200 (Lane 5) were separated on a 12% SDS-PAGE gel and stained with Coomassie brilliant blue R-250. B. Western blot analysis of purified His-tagged recombinant protein with mouse anti His-tag IgG.

**Additional file 3.** Purification of GST-tagged recombinant proteins. A. SDS-PAGE analysis of purified GST-tagged recombinant proteins. GST-tagged proteins of PF3D7_1238600 (Lane 1), PF3D7_0107200 (Lane 2), PF3D7_0305600 (Lane 3), PF3D7_1363500 (Lane 4) and PF3D7_0319200 (Lane 5) were separated on a 12% SDS-PAGE gel and stained with Coomassie brilliant blue R-250. B. Western blot analysis of purified GST-tagged recombinant proteins with an anti-GST tag IgG.
