# Supplementary figures and images for: Identification and characterization of DNA endonucleases in Plasmodium falciparum 3D7 clone
Source: Malar J. 2018 Jun 18;17:232. doi: 10.1186/s12936-018-2388-0 (PMC6006590; doi:10.1186/s12936-018-2388-0)

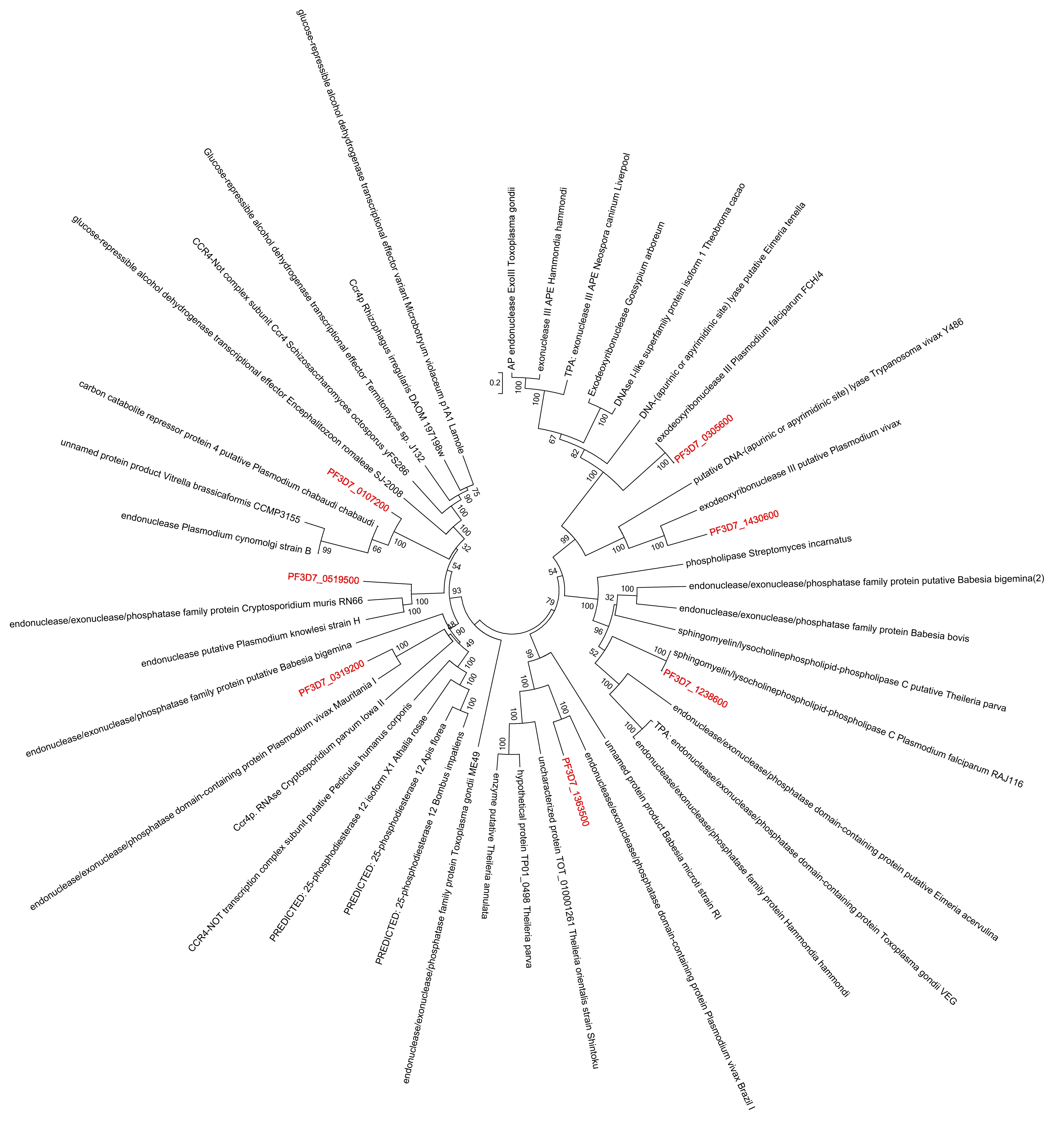

Supplement: Supplementary file 1 — Additional file 1. Phylogenetic analysis of seven P. falciparum 3D7 DNA endonucleases with homologous proteins of other species. Amino acid sequences were aligned using MEGA 6, and a phylogenetic tree was generated by the neighbour-joining method. The scale bar represents amino acid substitutions in the sequences and evolutionary distances. P. falciparum DNA endonucleases are highlighted in red. [file 12936_2018_2388_MOESM1_ESM.tif]

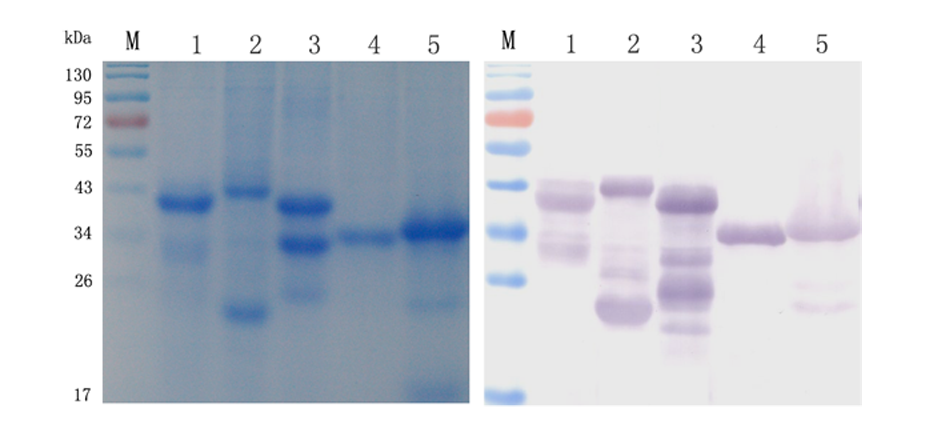

Supplement: Supplementary file 2 — Additional file 2. Purification of His-tagged recombinant proteins. A. SDS-PAGE analysis of purified His-tagged recombinant proteins. His-tagged proteins of PF3D7_1238600 (Lane 1), PF3D7_0107200 (Lane 2), PF3D7_0305600 (Lane 3), PF3D7_1363500 (Lane 4) and PF3D7_0319200 (Lane 5) were separated on a 12% SDS-PAGE gel and stained with Coomassie brilliant blue R-250. B. Western blot analysis of purified His-tagged recombinant protein with mouse anti His-tag IgG. [file 12936_2018_2388_MOESM2_ESM.tif]

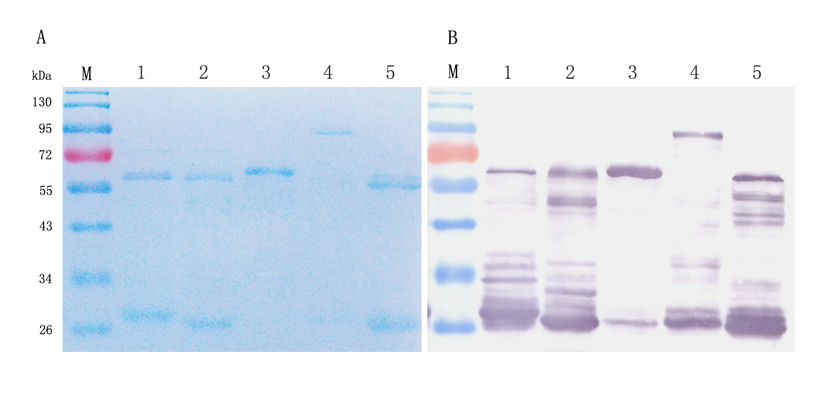

Supplement: Supplementary file 3 — Additional file 3. Purification of GST-tagged recombinant proteins. A. SDS-PAGE analysis of purified GST-tagged recombinant proteins. GST-tagged proteins of PF3D7_1238600 (Lane 1), PF3D7_0107200 (Lane 2), PF3D7_0305600 (Lane 3), PF3D7_1363500 (Lane 4) and PF3D7_0319200 (Lane 5) were separated on a 12% SDS-PAGE gel and stained with Coomassie brilliant blue R-250. B. Western blot analysis of purified GST-tagged recombinant proteins with an anti-GST tag IgG. [file 12936_2018_2388_MOESM3_ESM.tif]
